# Application of flipped classroom based on CDIO concept combined with mini-CEX evaluation model in the clinical teaching of orthopedic nursing

**DOI:** 10.1186/s12909-023-04200-9

**Published:** 2023-04-06

**Authors:** Xinyang Su, Huaxiu Ning, Fang Zhang, Li Liu, Xiaoling Zhang, Hongmei Xu

**Affiliations:** 1 Department of Spine surgery, Binzhou Medical University Hospital, Binzhou, China; 2 Department of Nursing, Binzhou Medical University Hospital, Binzhou, China; 3 School of Nursing, Binzhou Medical University, Binzhou, China

**Keywords:** CDIO teaching mode, Flipped classroom, Orthopedics, Mini-CEX, Nursing interns

## Abstract

**Background:**

After the COVID-19 epidemic, the state has paid more attention to the clinical teaching function of affiliated hospitals of colleges and universities. Strengthening the integration of medicine and education and improving the quality and effect of clinical practice teaching are critical challenges facing medical education. The difficulty of orthopedic teaching lies in the characteristics of a wide variety of diseases, strong professionalism, and relatively abstract characteristics, which affect the initiative, enthusiasm, and learning effect of nursing students. In this study, a flipped classroom teaching plan based on the CDIO (conceive–design–implement–operate) concept was constructed and practiced in the orthopedic nursing student training course to improve the effect of practical teaching, and it is convenient for teachers to implement more effective and targeted teaching in the flipped classroom of nursing education and even medical education in the future.

**Methods:**

Fifty undergraduate nursing students who practiced in the Orthopedics Department of a tertiary hospital in June 2017 were enrolled in the control group, while 50 undergraduate nursing students who practiced in the same department in June 2018 were enrolled in the intervention group. The intervention group adopted the flipped classroom teaching mode of the CDIO concept, whereas the control group adopted the traditional teaching mode. After finishing the department practice task, the students in the two groups completed the evaluation of theory, operation skills, independent learning ability, and critical thinking ability. They completed the evaluation of clinical practice ability in eight dimensions, including four processes of nursing procedures, humanistic care ability, and evaluation of clinical teaching quality for two groups of teachers.

**Results:**

After teaching, the clinical practice ability, critical thinking ability, autonomous learning ability, theoretical and operational performance, and evaluation of clinical teaching quality in the intervention group were significantly higher than those in the control group (all p < 0.05).

**Conclusion:**

The CDIO-based teaching mode can stimulate the independent learning ability and critical thinking ability of nursing interns, promote the organic combination of theory and practice, improve their ability to comprehensively use theoretical knowledge to analyze and solve practical problems, and improve teaching effectiveness.

## Background

Clinical teaching is a critical phase in nursing education that involves the transition from theoretical knowledge to practice. Effective clinical teaching can help nursing students master professional skills, strengthen their professional knowledge, and improve their ability to perform nursing activities. It is also the final stage in the transition of nursing students’ professional roles [1]. In recent years, many clinical teaching researchers have investigated problem-based learning (PBL), case-based learning (CBL), team-based learning (TBL), and situational learning in clinical teaching, scenario simulation teaching, and other teaching methods. However, different teaching methods have their own merits and shortcomings regarding the teaching effectiveness of the practice link, but they have not achieved the integration of theory and practice [2].

Flipped classroom refers to a new teaching mode in which students learn diversified learning materials independently before class with the help of a certain information platform, complete homework in the form of “cooperative learning” and other forms in class, and at the same time, teachers answer questions and provide individualized help [3]. The American New Media Alliance noted that the flipped classroom readjusts the time inside and outside the classroom and transfers students’ learning decisions from teachers to students [4]. Valuable time in the classroom in this teaching mode allows students to focus more on active problem-based learning. Deshpande [5] carried out a flipped classroom in the teaching of doctor assistant education, and concluded that flipped classroom can improve students’ learning enthusiasm and academic performance, and shorten the class time.Khe Foon HEW and Chung Kwan LO [6] examined the findings of comparative articles through a meta-analysis to summarize the overall effects of teaching with the flipped classroom approach, suggesting that the flipped classroom approach in health profession education significantly improves student learning compared with traditional teaching methods. Zhong J [7] compared the effectiveness of blended learning between a flipped virtual classroom and a flipped physical classroom on students’ knowledge learning and discovered that in a flipped classroom with a blended learning process of histology, enhancing the quality of online learning boosts student satisfaction and improves knowledge learning. Based on the above research, it is found that in nursing education, most scholars study the impact of flipped classrooms on classroom teaching effects and believe that flipped classroom teaching can improve nursing students’ academic performance, autonomous learning ability, and classroom satisfaction.

Therefore, to promote nursing students to internalize systematic professional knowledge in their hearts and externalize it in practice to improve their clinical practice ability and comprehensive quality, it is urgent to explore and construct a novel teaching method.CDIO (conceive–design–implement–operate) is an engineering education model developed in 2000 by four universities, including the Massachusetts Institute of Technology and the Royal Swedish Institute of Technology. It is an advanced engineering education model that allows nursing students to learn and acquire competencies in an active, hands-on, and organically linked way among courses [8, 9]. On the subject of teaching, this model emphasizes "student-centered” students participate in the conception, design, implementation, and operation of the project and transform the theoretical knowledge they have learned into a tool for solving problems. Several studies have revealed that the CDIO teaching mode is conducive to improving the clinical practice ability and comprehensive quality of nursing students, enhancing the interaction between teachers and students, improving the teaching effect, and playing a certain role in promoting the informatization and optimization reform of teaching methods. Currently, it is widely used in the field of applied talent training [10].

With the transformation of the global medical model, the demand for health is increasing, and higher burdens of responsibility are also placed on medical personnel. The ability and quality of nurses are directly related to the quality of clinical care and patient safety. Recently, cultivating and evaluating the clinical ability of nursing staff have become hotspots in the nursing field [11]. Therefore, an objective and comprehensive evaluation method with high reliability and validity is vital for medical education research. Mini-clinical evaluation exercise (mini-CEX), an assessment method for evaluating the comprehensive clinical ability of medical students, has been widely used in the field of multidisciplinary medical education in China and abroad. It has gradually emerged in the field of nursing [12, 13].

At present, several studies have reported the application of the CDIO model, flipped classroom, and mini-CEX in nursing education. Wang Bei [14] discussed the effect of the CDIO model on improving special nursing training oriented to the nursing needs of the new coronary pneumonia disease. The results demonstrated that using the CDIO teaching model to carry out special nursing training on the new coronary pneumonia disease is conducive to the nursing staff to better master specialized nursing training skills and related knowledge, comprehensively improving their comprehensive nursing ability. Liu Mei [15] and other scholars discussed the application of a team-based teaching method combined with a flipped classroom in the training of orthopedic nurses, and the results depict that the teaching mode can effectively improve the core competencies of orthopedic nurses such as understanding and application of theoretical knowledge, teamwork, critical thinking and scientific research. Li Ruyue [16] researched the application effect of the improved Nursing Mini-CEX in the standardized training of new surgical nurses and found that teachers can find their weaknesses in clinical teaching or work by using Nursing Mini-CEX to evaluate the whole process of assessing nurses and giving real-time feedback. In the link between self-supervision, self-reflection, absorbing the bright spots in the assessment of nurses’ work, adjusting the teaching plan, and further improving the quality of clinical teaching, students can improve the comprehensive ability of surgical clinical nursing, test the actual clinical processing ability in different situations, deeply understand and improve nursing work, and cultivate strong professional ethics and communication skills.However, no study has reported the application of a CDIO concept-based flipped classroom combined with mini-CEX evaluation model in the teaching of orthopedic nursing students. The author employed the CDIO model in the design of the orthopedic nursing student training course, constructed a flipped classroom based on the CDIO concept and combined the mini-CEX evaluation model, realized the“three-in-one”training mode of knowledge, ability, and quality, promoted continuous improvement in teaching quality, to provide a reference for practical teaching in teaching hospitals.

## Materials and methods

### Study design

A prospective controlled experimental design was used in this study.

### Participants

To facilitate the implementation of the course, convenience sampling was used to select the 2017 and 2018 undergraduate nursing students who practiced in the Orthopedics Department of a tertiary hospital as the research objects. Since there were 52 interns at each level, the sample size was 104. Four students did not participate in full clinical practice.Fifty undergraduate nursing students who practiced in the Orthopedics Department of a tertiary hospital in June 2017 were enrolled in the control group, including 6 males and 44 females; aged 20 to 22 (21.30 ± 0.60) years old; fifty undergraduate nursing students who practiced in the same department in June 2018 were enrolled in the intervention group, including 8 males and 42 females, aged 21 to 22 (21.45 ± 0.37) years old. All subjects gave informed consent. Inclusion criteria: (1)Orthopedic intern nursing students with a bachelor’s degree. (2)Informed consent and voluntary participation in this study. Exclusion criteria: Those who cannot fully participate in the clinical practice. There was no significant difference in the general data of the two groups of trainee nursing students (p > 0.05), which was comparable.

### Training methods

Both groups had a four-week-long clinical practice, and all courses were completed in the orthopedic department.During the observation period, there were ten batches of nursing students, with five students in each batch. The teaching was completed in accordance with the nursing student practice syllabus, which included two parts: theory and skill operation. The two groups of teachers had the same qualifications, and the teaching nurse was responsible for the quality control of teaching.

### Control group

The control group adopted the traditional teaching method. In the first week of admission, the teaching commenced on Monday. The teacher taught the theory on Tuesday and Wednesday, and the operation training was concentrated on Thursday and Friday. From the second to the fourth week, each teacher was responsible for one nursing student to conduct random teaching in the department. In the fourth week, the assessment and evaluation were completed three days before leaving the department.

### Intervention group

As stated earlier, the author adopted the flipped classroom teaching method based on the CDIO concept, described as follows.

The first week of teaching was the same as that of the control group; the second to fourth weeks of orthopedic perioperative teaching adopted a flipped classroom teaching plan based on the CDIO concept, with a total of 36 class hours. The conception and design part was completed in the second week, and the implementation part was completed in the third week. The operation was completed in the fourth week, and the assessment and evaluation were completed three days before leaving the department. Specific allocations of class hours are presented in Table 1.

#### Step one: form a teaching team

A teaching team consisting of one head nurse in charge of teaching, eight orthopedic teachers, and one non-orthopedic CDIO nursing expert was established. The head nurse organized teaching group members to study and master the CDIO outline and standards, the CDIO workshop manual, and other related theories and specific implementation methods (for no less than 20 hours) and consulted experts at any time on challenging problems in theoretical learning. In accordance with the requirements of Adult Nursing and the syllabus of practice, the teaching team formulated teaching objectives, led teaching plans, and prepared lessons uniformly.

#### Step two: setting teaching objectives

According to the internship training syllabus, referring to the CDIO talent training syllabus and standards [17], and combining with the characteristics of orthopaedic nursing teaching, the teaching objectives of the internship nursing students were set in three dimensions, namely knowledge objectives (mastering basic professional knowledge and related system processes, etc.), ability objectives (enhance professional basic skills, critical thinking ability and autonomous learning ability, etc.) and quality objectives(establish correct professional values and humanistic care spirit, etc.).Among them, the knowledge objectives correspond to the technical knowledge and reasoning of the CDIO syllabus, which corresponds to the personal ability, professional ability, and attitude of the CDIO syllabus, and the quality objectives correspond to the interpersonal skills of the CDIO syllabus: teamwork and communication.

#### Step three: develop a teaching plan

After two rounds of meetings, the teaching team discussed the flipped classroom nursing practice teaching plan based on the CDIO concept, divided the teaching into four stages, and determined the goals and outlines, as summarized in Table 1.

**Table 1:** The flipped classroom teaching scheme based on the CDIO concept

Step	Teaching objectives	Teaching approach	Teaching evaluation	Class hours
Conceive	1.Master professional knowledge 2.Cultivate independent learning ability	1.Before the training, teachers conceived perioperative period cases of orthopedic. The relevant knowledge and skills, key points, and difficult points involved in the cases, and related micro-lecture videos were distributed to nursing students through the network platform. 2. Nursing students independently consulted the literature according to case problems and difficult points.	1.Theoretical examination 2. Class report 3.Self-directed learning ability assessment	8
Design	1. Cultivate professional systems thinking 2. Enhance communication and teamwork skills 3. Enhance critical thinking skills	1. Nursing students were free to form unit groups. They found theoretical basis or materials for solving case problems in their spare time during practice. Teachers guided nursing students in designing specific scenarios for scenario simulation. 2.Discussed in groups and improved the specific plan of perioperative care.		4
Implement	1. Exercise basic skills of specialist practice 2. Cultivate the spirit of humanistic care 3. Exercise emergency and coordination skills	1.Nursing students reported cases in groups according to the relatively well-conceived and designed nursing plan. After the report was over, other group members and teachers discussed and commented on the report group to further improve the nursing plan. 2.The group leader led the group members to simulate the overall nursing process, and the teacher guided the nursing students to deepen their understanding and mastery of theoretical knowledge in the process of simulated practice.	1.Project mutual evaluation and comment 2.Operational assessment 3.Assessment of critical thinking skills	16
Operate	1. Establish correct professional responsibilities and values 2. Develop the ability to discover and solve problems	1.Nursing students conducted relevant professional theoretical assessments after the actual operation of the practical project. 2. At the end of the assessment, teachers made comments and summaries.	1.Clinical practice ability assessment 2.Satisfaction evaluation	8

#### Step four: implement the CDIO teaching model

##### Project conceive

After analyzing the nursing work on orthopedic ailments, the teacher conceived the cases of common diseases and frequently occurring orthopedic diseases. Take the nursing program for patients with lumbar disc herniation as an example: Patient Zhang Moumou (male, 73 years old, 177 cm tall, and 80 kg in weight) complained of “low back pain with left lower limb numbness and pain for 2 months” and was admitted to the outpatient clinic As a responsible nurse: (1) Please systematically take the medical history based on the knowledge you have learned and judge what happened to the patient; (2) Select the systemic and specialist evaluation approach according to the condition and propose the inspection items that require further evaluation; (3) When making a nursing diagnosis, find the basis in the case; write out the targeted nursing measures related to the patient; and (4) Discuss the problems existing in the patient’s self-management and the current method and content of the patient’s discharge follow-up. Post the student case and task list two days before the class. The task list for this case is as follows: (1) Review and consolidate theoretical knowledge on the etiology and clinical manifestations of lumbar disc herniation; (2) Make a targeted nursing plan; (3) Design this case according to clinical work to execute two main scenario simulation learning projects of pre- and post-operative nursing. Nursing students independently previewed the course content with case questions, consulted relevant literature and databases, and completed the self-learning tasks in the form of WeChat group solitaire check-in.

##### Project design

Students freely formed unit teams,the team chose a team leader, and the team leader was responsible for the division of labor and coordination of the project. The pre-class team leader was responsible for allocating the four contents of case introduction, nursing procedure implementation, health education, and disease-related knowledge to each team member. Students sought theoretical basis or materials for solving case problems in their spare time during the internship, discussing in teams and perfecting specific project plans. While designing the project, the teacher assisted the team leader in assigning team members to be responsible for sorting out relevant knowledge points, designing and producing projects, demonstrating and revising projects, and instructing nursing students to integrate professional-related knowledge points into the design and experience the knowledge of each module. The difficulties and key points of this group were sorted out and designed to implement the scenario simulation implementation plan for this group. Teachers also organized a bedside nursing ward round demonstration at this stage.

##### Project implementation

Students made project reports in teams. After the report was over, other group members and teachers discussed and commented on the report group to further improve the nursing plan. The team leader led the team members to simulate the overall nursing process, and the teacher guided the students to deepen their understanding and construction of theoretical knowledge and develop critical thinking ability through learning the dynamic changes of the disease during the simulation practice.The practical operations that need to be completed in the development of specialist diseases were completed under the guidance of teachers, who commented on and led nursing students to complete bedside practices to achieve the integration of knowledge points and clinical practices.

##### Project operation

After the assessment of each group, the teacher made comments, noted the strengths and weaknesses of the members of each group in the process of content arrangement and skill operation, and continuously improved the nursing students’ understanding of the teaching content.After the project, teachers completed the analysis of teaching quality and optimized the curriculum according to the assessment results of nursing students and their evaluation of teaching.

### Observation indicators

#### Theory and operational assessment

Nursing students completed the theory and operation examinations after practical teaching. Theoretical intervention questions were set by the instructor.The intervention papers were divided into two sets (A and B), and one set was randomly selected for the intervention. The intervention questions were divided into two parts, specialist theoretical knowledge and case analysis, each with 50 points, for a total of 100 points. Nursing skills assessment students randomly selected one of them, including axial turning technique, good limb placement technique for patients with spinal cord injury, the technique of using air pressure therapy apparatus, the technique of using CPM Joint rehabilitation machine, etc. The full score is 100 points.

#### Self-directed learning ability evaluation scale

In the fourth week, the assessment of Self-directed learning ability evaluation scale was conducted three days before leaving the department.The self-directed learning ability evaluation scale developed by Zhang Xiyan [18] was used, including learning motivation (8 items), self-management ability (11 items), learning cooperation ability (5 items), and information literacy (6 items).Each item was scored on a 5-point Likert scale, that is, from “completely inconsistent” to “completely consistent”, with 1 to 5 points. The total score is 150 points, and the higher the score, the stronger the autonomous learning ability. The scale Cronbach’s alpha coefficient was 0.822.

#### Critical thinking ability assessment scale

In the fourth week, the assessment of Critical Thinking Ability Assessment Scale was conducted three days before leaving the department.The Chinese version of the Critical Thinking Ability Assessment Scale translated by Meici [19] was used. It has seven dimensions, including finding the truth, open mind, analytical ability, and systematization ability, with 10 items for each dimension. A 6-level scoring was used, that is, from “strongly disagree” to “strongly agree,” with 1 to 6 points, respectively, and negative statements were scored in reverse, with a total score of 70 to 420 points. A total score of ≤ 210 indicates negative performance, 211–279 indicates neutral performance, 280–349 indicates positive performance, and ≥ 350 indicates strong critical thinking ability. The scale Cronbach’s alpha coefficient was 0.90.

#### Assessment of clinical practice ability

In the fourth week, the assessment of clinical practice ability was conducted three days before leaving the department.The mini-CEX scale used in this study was adapted from YiJing [20] in accordance with mini-CEX, with 1–3 points for not meeting the requirements, 4–6 points for meeting the requirements, and 7–-9 points for good. Nursing students completed their training after the completion of the specialist practice. The Cronbach’s alpha coefficient of the scale was 0.780, and the split-half reliability coefficient was 0.842, indicating good reliability.

#### Evaluation of teaching quality

In the fourth week,a teacher-student symposium and the evaluation of teaching quality were conducted the day before leaving the department.The teaching quality evaluation table was designed by Zhou Tong [21], including five aspects: teaching attitude, teaching content, teaching method, teaching effect, and teaching characteristics. A 5-point Likert scale was used. The higher the score, the better the teaching quality. Completed after completion of the specialist internship. The questionnaire has good reliability, the Cronbach’s alpha coefficient of the scale was 0.85.

### Data analysis

SPSS 21.0 statistical software was used to analyze the data. Measurement data were expressed as mean ± standard deviation ($\overline X \pm S$), and group t intervention was used for comparison between groups. Enumeration data were expressed as cases (%), and comparison was made using chi-squared intervention or Fisher’s exact intervention. p-value < 0.05 corresponded to a statistically significant difference.

## Results

The comparison of the theoretical and operational intervention scores of the two groups of nursing interns is shown in Table 2.

**Table 2:** Comparison of the theoretical and operational intervention scores of the two groups of nursing students ($\overline X \pm S$)

Group	Number of subjects	Theoretical assessment results	Operational assessment results
Control group	50	91.48 ± 3.63	91.86 ± 1.87
Intervention group	50	95.94 ± 2.53	96.72 ± 1.47
t-value		−7.128	−14.425
p-value		< 0.001	< 0.001

Comparison of scores of independent learning ability and critical thinking ability of nursing interns in the two groups are shown in Table 3.

**Table 3:** Comparison of scores of independent learning ability and critical thinking ability of nursing students between the two groups ($\overline X \pm S$)

Group	Number of subjects	Self-learning ability	Critical thinking skills
Control group	50	101.76 ± 4.40	235.66 ± 15.14
Intervention group	50	112.32 ± 4.85	242.62 ± 14.46
t-value		−11.406	−2.351
p-value		< 0.001	< 0.001

Comparison of the clinical practice ability assessment of nursing interns in the two groups.The clinical nursing practice ability of the students in the intervention group was significantly better than that in the control group, and the difference was statistically significant (p < 0.05), as shown in Table 4.

**Table 4:** Comparison of clinical practice ability of the two groups ($\overline X \pm S$)

Group	Number of subjects	Nursing consultation	Nursing physical examination	Nursing diagnosis	Nursing measures	Health advisory	Organizational effectiveness	Humanistic care	Overall evaluation
Control group	50	5.64 ± 1.17	4.96 ± 1.18	5.20 ± 1.40	5.32 ± 1.33	5.58 ± 1.20	6.14 ± 1.23	5.60 ± 1.09	5.12 ± 1.10
Intervention group	50	6.86 ± 1.34	5.6 ± 1.05	6.24 ± 1.29	6.66 ± 1.41	7.44 ± 1.25	7.66 ± 1.15	7.46 ± 1.15	6.92 ± 0.97
t-value		−4.842	−2.869	−3.868	−4.888	−7.606	−6.243	−8.321	−8.696
p-value		< 0.05	< 0.05	< 0.05	< 0.05	< 0.05	< 0.05	< 0.05	< 0.05

The teaching quality evaluation results of the two groups showed that the total score of teaching quality in the control group was 90.08 ± 2.34 points, and the total score of teaching quality in the intervention group was 96.34 ± 2.16 points, and the difference was statistically significant (t = − 13.900, p < 0.001).

## Discussion

The development and progress of medicine require medical talents to conduct sufficient practice accumulation. Although there are many simulation and simulation training methods, they cannot replace clinical practice, which is directly related to the level of future medical talents to treat diseases and save lives. After the new crown epidemic, the state paid more attention to the clinical teaching function of the affiliated hospitals of colleges and universities [22]. Strengthening the integration of medicine and education and improving the quality and effect of clinical practice teaching are critical challenges facing medical education. The difficulty of orthopedic teaching lies in the characteristics of a wide variety of diseases, strong professionalism, and relatively abstract characteristics, which affect the initiative, enthusiasm, and learning effect of nursing students [23].

The flipped classroom teaching method of CDIO teaching concept integrates teaching content with the process of teaching, learning, and doing. It changes the structure of the teaching classroom and makes nursing students the backbone of the teaching scene. In the teaching process, teachers guide nursing students to independently consult relevant materials for difficult nursing problems in typical cases [24]. Research has shown that CDIO involves the task designing and activity of clinical practice teaching. The design provides detailed guidance, which closely integrates the consolidation of specialized knowledge points and the cultivation of hands-on operation ability and finds problems during simulation, which is beneficial for nursing students to improve their independent learning ability and critical thinking ability in self-study and guidance. The results of this study showed that the scores of autonomous learning ability and critical thinking ability of nursing students in the intervention group were significantly higher than those in the control group after four weeks of training (both p < 0.001). This is consistent with Fan Xiaoying’s research results [25], who studied the application effect of CDIO combined with the CBL teaching method in internal medicine nursing teaching. This teaching method can significantly improve the critical thinking and autonomous learning abilities of interns.In the conception stage, the teacher first handed out the difficult points in the classroom case to the nursing students. The nursing students then learned relevant information independently through the micro-lecture videos and actively searched for relevant materials to further enrich their understanding of orthopedic nursing work. In the design process, nursing students, under the guidance of their teachers, relied on cases and exercised teamwork and critical thinking skills in group discussions. In the realization stage, the teachers took the actual disease perioperative nursing as an opportunity and used the scenario simulation teaching method to guide the nursing students to execute scenario drills under group cooperation to familiarize themselves with and find out the problems in the nursing work. At the same time, in teaching real cases, nursing students could learn the key points of pre- and post-operative nursing so that they could clearly understand that each link of perioperative nursing is an important factor in patients’ post-operative rehabilitation. In the operation link, teachers guided nursing students to master theories and skills in practice. At the same time, they learned to observe changes in the condition and think about possible complications in actual cases so that they no longer memorized various nursing procedures by rote and helped nursing students. In the process of construction and implementation, the teaching-related content was organically integrated. In this participatory, interactive, and experiential learning process, nursing students’ autonomous learning ability and learning enthusiasm were well mobilized, and their critical thinking ability was improved.Scholars used a Design Thinking (DT)-Conceive-Design-Implement-Operate (CDIO) engineering design framework in a fipped web programming course to develop students’ learning achievement and computational thinking (CT) abilities, and the results displayed that the students significantly improved their learning achievement and computational thinking ability [26].

In this study, nursing students were guided to participate in the whole process according to the process of“asking questions- conception- design- implementation -operation-summary.”First, teachers published cases, designed problems, guided nursing students to conceive, and designed clinical situations. Then, based on group collaboration and independent thinking, supplemented by the teacher answering questions, students put forward solutions to problems to complete data collection, situational exercises, and finally complete bedside practice. The results of the study showed that the scores of the theoretical knowledge assessment and operation skills assessment of nursing students in the intervention group were better than those in the control group, and the differences were statistically significant (p < 0.001),and this was consistent with the findings of related research [27, 28]. The reason for the analysis is that in the CDIO model, the disease knowledge points with higher morbidity are first selected, and second, the difficulty of item setting is in line with the fundamental level. In this model, after learning the practical content, students complete the project task book as needed, check the relevant content again, and discuss the tasks with the group members to realize the digestion and absorption of the learning content and summarize the new knowledge and the old knowledge in a new way. The mastery of knowledge is improved.

This study shows that through the application of the CDIO clinical teaching model, the nursing students in the intervention group were better than the nursing students in the control group in terms of nursing consultation, physical examination, determination of nursing diagnosis, implementation of nursing measures, and humanistic care. Furthermore, statistically significant differences were found in each dimension between the two groups (p < 0.05), which was similar to the results of Hongyun [29]. Zhou Tong [21] studied the application effect of the teaching mode under the guidance of the conception-design-implementation-operation concept (CDIO) in the clinical practice teaching of cardiovascular specialist nursing and found that the students in the experimental group who adopted the CDIO clinical practice teaching method were significantly better than those who adopted the traditional teaching method in the eight dimensions of nursing procedures, humanistic care ability, and overall.It may be because in the learning process, nursing students no longer passively accept knowledge but use their various abilities to acquire knowledge. Team members give full play to team spirit to integrate learning resources and repeatedly report, practice, analyze, and discuss clinical practical nursing problems, cognition from the shallower to the deeper, and pay more attention to the specific content of the cause analysis of health problems, the formulation of nursing goals, and the feasibility analysis of nursing measures. Teachers guide and demonstrate in the discussion to form a cyclic stimulus of perception–practice–response to guide nursing students to complete a meaningful learning process and improve the clinical practice abilities of nursing students, improve the interest and efficiency of learning, and continuously improve the ability to learn from theory to practice while completing the internalization of knowledge.

The application of the CDIO-based clinical teaching scheme improved the quality of clinical teaching. The research results of Ding Jinxia [30] and others suggest a correlation between various dimensions of learning motivation, autonomous learning ability, and the effective teaching behavior of clinical teachers.In this study, with the development of CDIO clinical teaching, clinical teachers received several specialized trainings, updated teaching concepts, and improved their teaching abilities. Second, it enriches the clinical teaching cases and teaching content of cardiovascular nursing and grasps the orderliness and operability of the teaching mode from a macro perspective, which is conducive to students’ understanding and memory of the course content. The feedback after each lecture can promote clinical teachers’ self-awareness and urge clinical teachers to reflect on their own skills, professional level and humanistic accomplishment to truly achieve mutual teaching and improve the quality of clinical teaching. The results showed that the teaching quality of clinical teachers in the intervention group was better than that in the control group,which was similar to Xiong Haiyan’s research results [31].

### Limitations and suggestions

Although the findings of this study are valuable for clinical teaching, there are still many limitations in our research. First, the use of convenience sampling may limit the generalization of these findings, and we also had a limited sample for one tertiary hospital. Second, the training is conducted only four weeks, and the critical thinking skills of nursing interns need more time to develop. Third, in this study, the patients used in Mini-CEX are real patients who have not been trained, the quality completed by the nursing interns may vary from patient to patient. These are the main issues that limit the findings of this study. Future research is needed to expand the sample size, and increase the training of clinical teachers, unify the design standards of teaching cases. Longitudinal studies should also be conducted to examine whether the flipped classroom based on the concept of CDIO can foster the comprehensive ability of nursing students over a long period.

## Conclusions and implications

This study developed the CDIO model in designing an orthopedic nursing student training course, constructed a flipped classroom based on the CDIO concept, and combined the mini-CEX evaluation model. The results indicated that the flipped classroom based on the concept of CDIO improved students’ independent learning ability, critical thinking ability, and clinical practice abilities while improving the quality of clinical teaching. This teaching method was more credible and effective than the traditional lectures. It can be concluded that study results may have implications for medical education. The flipped classroom, based on the concept of CDIO, has significant potential to prepare students for performing clinical work by focusing on teaching, learning, and doing, which closely integrates the consolidation of specialized knowledge points and the cultivation of hands-on operation ability. Because of the importance of involving students in learning and practicing for positive, it is proposed that the CDIO-based clinical teaching mode can be used in medical education considering all aspects. This method can also be recommended as an innovative and student-centered method for clinical teaching. In addition, the results can be extremely helpful to policymakers and academics in developing strategies for improving medical education.

## Data Availability

The datasets used and/or analyzed during the current study are available from the corresponding author on reasonable request.
